# Caregiving: a risk factor of poor health and depression among informal caregivers in India- A comparative analysis

**DOI:** 10.1186/s12889-022-14880-5

**Published:** 2023-01-06

**Authors:** Ruchira Chakraborty, Arjun Jana, Viraj Mahesh Vibhute

**Affiliations:** International Institute for Population Sciences, Mumbai, 400088 India

**Keywords:** Caregiver, Depressive symptoms, Self-rated health, CES-D Scale, LASI

## Abstract

**Background:**

In an ageing society, the provision of long-term care is the prime need. In Indian cultural setting, family members are the informal, albeit primary caregivers to the elderly. Caregiving demands intense emotional and financial involvement. While taking care of elderly persons’ health and wellbeing, these family members, acting as informal caregivers, may themselves become vulnerable to poor health due to additional stress and burden. Using a nationally representative survey, the study tried to identify how health condition varies within caregivers and a comparative analysis of how in similar socio-economic background health condition varies between caregivers and non-caregivers.

**Method:**

The data, used for the analysis, is taken from Longitudinal Ageing Study in India (LASI), Wave I. Both descriptive and multivariable regression analysis are done in different models along with interaction effect of caregiving to understand the difference in health status between caregiver and non-caregivers.

**Results:**

Nearly 29% and 11% of the informal caregivers, reported to have depressive symptoms and poor self-rated health (SRH), respectively. Almost half of the caregivers, who provide care for more than 40 h a week, are diagnosed to have depressive symptoms. They are also at higher risk of having depressive symptoms (AOR 1.59 CI 1.16–2.18) and poor SRH (AOR 1.73 CI 1.11–2.69) than those who invest less than 40 h in a week. In almost every socio-economic condition, caregivers are at a higher risk of having depression and poor health than non-caregivers. Caregivers, who are widowed, live in rural areas or are not satisfied with current living arrangement are more vulnerable to have depressive symptoms. On the other hand, caregivers of age 45–59 years, widowed, male and who live only with their children with spouse absent, have almost 2 times higher odds of poor SRH than non-caregivers.

**Conclusion:**

Caregivers are more susceptible to depression and poor self-rated health compared to non-caregivers irrespective of their socio-economic characteristics, only the magnitude of vulnerability varies.

**Supplementary Information:**

The online version contains supplementary material available at 10.1186/s12889-022-14880-5.

## Background

With rising life expectancy and health transition, the global burden of diseases increased up to 23% due to chronic and long-term health impairments among the older adults [1]. India, on verge of having an ageing society, would be facing the need for care services in near future with increasing share of chronic disease burden among older adults [2]. For palliative care services, the need for in-house care would be more than institutional care services. In traditional Indian as well as other South and South East Asian societies, informal caregiving is more popular where family is the primary source of caregiver where filial piety is deeply rooted in social and cultural dimensions [3, 4]. Since long this cultural system encourages and bound co-residing family members to be the informal caregivers. In Indian households, spouse is the primary person to provide care along with daughter-in-law from the younger generation. Thus, caregiver’s age has a very broader range; older adults can be care-receivers as well as caregivers. On the other hand, formal or paid care services for the elderly induces higher economic burden for the care recipients and to the family specially in absence of proper social security system [5–8].

Informal caregivers are persons, who provide day-to-day instrumental, financial, social and emotional support to a family member or a closely related person in need but not as occupation [9, 10]. Thus, caregiving is a burdensome, unpaid work and somewhat challenging task for the caregivers. It has been found to have a long-term effect on caregivers’ physical or emotional health along with financial stress, anxiety and social isolation [11–15]. Studies in United States of America, also suggest, informal caregiving to be an important factor contributing towards developing depressive symptoms as well as poor physical health which may lead to suffering from impaired immune function and increased risk of mortality [16, 17]. Relationship quality between the caregiver and care recipient is an immediate determinant of caregiving role to be burdensome or not. If the role of caregiving gets overloaded and conflict arises, that influences the caregiver’s burden indirectly [18]. Gender differentials in burden of caregiving suggests that, the role of women in household activities and economic participation conflict with the role of caregiving and thus women caregivers used to have poorer relationship quality with care recipient which gradually works as a factor for developing poor physical and mental health [18, 19]. Caregiver’s general health also gets heavily affected by the type of disease afflicting the care recipient. Providing care to dementia or Alzheimer’s patient shows significantly higher prevalence of depressive symptoms, especially among the female caregivers due to severe stress, inability to communicate and difficulty in coping with the behavioral problems of the care recipient [8, 20].

Though psychological distress afflicting caregivers is widely acknowledged, the physical aspect of caregivers’ health has been less explored. Meta-analysis by Pinquart & Sorensen (2003) suggested that, the caregivers have lower levels of subjective well-being and poorer physical health than non-caregivers in United States and they were termed as “hidden patients”. Poor physical health among caregivers is highly associated with poor mental health, lack of sleep or poor sleeping pattern, poor diet, anxiety and stress for increased medical expenditure for the care recipient etc. [21–24].

On the contrary, in some cases the effect of caregiving has been identified to have positive impact on mental health or does not add on stress when there exists a closely knitted emotional bond between caregiver and care recipient [25–28] but it varies widely with type of care needed and health condition of the recipient. Even caregivers under specific situations and destressed conditions reported to have 18% reduced risk of all-cause mortality in comparison to non-caregivers [29]. Emotional investment and responsibility towards a care recipient with disability, has been found to affect the mental health condition of the caregivers the most [30]. But even if the caregivers value the role of caregiving and not only be obliged to do it, they require external support to lead their own quality life [29].

In Indian context the literatures related to caregiving are mostly focused to intergenerational gender wise role of caregiving or burden of caregiving [31, 32] in absence of co-residence [18, 33–35]. Neither health status of caregivers was focused nor ever compared with non-caregivers. Hence, this study has tried to explore whether caregivers’ health condition varies according to their socio-economic condition or according to the type of care provided or with relationship to care-receiver, using nationally representative survey data. The study also tried to fill the research gap by providing a comparative analysis of caregiver’s and non-caregiver’s health condition under similar socio-economic situations. To understand health,depressive symptoms are taken as a component of mental health and self-rated health as a component of overall health condition. Depressive symptoms have been measured through well accepted CES-D scale and it does not solely depend on self-reporting, on the other hand self-rated health (SRH) tries to capture the overall health condition or say health expectations from the point of view of the respondents. To compare health condition of the caregivers with that of non-caregivers, the characteristics unique to only caregivers e.g., caregivers’ burden, are exempted.

## Methods

### Data source

The data for the study, has been taken from Longitudinal Ageing Study in India [36] (LASI wave I) for a cross-sectional analysis. It is a nationally representative data for adults aged 45 and above, conducted by International Institute for Population Sciences in collaboration with Harvard T.H Chan School for Public Health and University of Southern California in the 2017–2018. LASI is India's first comprehensive survey, which includes demographics, household economic status, chronic health conditions, symptom-based health conditions, functional health, mental health (cognition and depression), biomarkers, health insurance and healthcare utilisation, family and social networks, social welfare programmes, work and employment, retirement, satisfaction, and life expectations. Regarding inhouse caregiving, information related to type of care, relationship with the caregiver, time spend on caregiving and burden of care is available in the data set. The survey contains well-developed measures for evaluating the impact of policy changes on health outcomes among India's older population. Respondents selected for the survey are above 45 years of age but some information about their spouse is also collected irrespective of the spouse’s age. The total number of households covered under the survey is 42,949. 72,250 individuals were interviewed among which 31,464 people are of age 60 and above. In this analysis all the respondents are taken into account irrespective of their age. For the dependent variables depressive symptoms as well as poor self-rated health, those who did not respond to the questions are considered as missing values and excluded from the analysis; (*n* = 1784) & (*n* = 935) respectively. After considering all the missing values from the explanatory variables the final sample size for the study is 67749.

### Variables

#### Outcome variables

##### Depressive symptoms

In this research, CES-D scale (Centre for Epidemiologic Studies Depression) has been used to identify presence of short-term depressive symptom among respondents [15, 16, 37]. It is a 10 item self-reported scale to identify presence of depressive symptoms. In this survey the reference period of respondent’s depressive symptoms is restricted within past one week of the survey period. The scale comprised of 10 types of separate symptoms; among them 7 are classified as “negative symptoms” (i.e., trouble concentrating, feeling depressed, feeling tired, afraid of something, feeling alone, bothered easily and feeling everything is an effort) and 3 items are classified as “positive symptoms” (i.e., satisfied, feeling hopeful about future and happy). All these questions have 4 sets of responses i.e., “Rarely or Never” (less than 1 day), “Sometimes” (1 or 2 days), “Often” (3 or 4 days), “Most or all of the time” (5–7 days). The first two options (rarely/never and sometimes) are scored as “0” and the other two options (often and most/all of the time) are scored as “1” for the negative symptoms. Reverse is done for the positive symptoms where rarely, never and sometimes are scored as “1” and often and most/all of the times are scored as “0”. The score ranges between 0–10. Having a score of 4 and above is considered to have depression [36]. The value of Cronbach’s Alpha for this 10 item CES-D scale is 0.80 which ensures high reliability in capturing depressive symptoms.

##### Self-rated health

In a set of questions respondents were asked to rate their health and the options given were- very good, good, fair, poor and very poor. For analysis of Self-rated Health, this variable is made binary where very good, good and fair are reclassified as “good health (1)” and poor and very poor are clubbed as “poor health (0)”. Self -rated health (SRH) is found to be a reliable measure of overall health condition and has previously been used in different studies [14, 22].

#### ﻿Explanatory variables

##### Hours spent in caregiving

Adult caregivers of the household were asked a few questions related to the time spending for caregiving process- “How often do you take care of the family member/outside the family?” and “For how many hours do you provide care in the last week?”. In the analysis, hours spent in caregiving is classified into two sections; less than 40 h a week is termed as “part time” and more than 40 h is termed as “full time”.

##### Type of care activities

Type of activities done as a part of providing care has been classified into 5 types; care for Activities in Daily Living (ADL); Instrumental Activities in Daily Living (IADL); Managing medications or changing bandages or accompanying the person to hospital, termed as Medical Care, keep watch on them or spending time with them, termed as Social or Emotional Care, and providing Financial Support.

##### Relationship to the care recipient

Relationship between the primary caregiver and care recipient is classified into 7 groups; Spouse/partner, Parents, Parents-in-law, Brothers/Sisters, Children, Other relatives and not related. The options provided could not be gender distinguished due to data limitation.

#### Covariates

Socio-economic and demographic profile of the all respondents is taken into account to carry out a comparative analysis between caregiver’s and non-caregiver’s health condition. Respondent’s demographic profile includes Age (< 45 years, 45–59 years, 60–69 years and above 70 years); Sex (Male or Female), Marital Status (currently married, widowed and Others), Education (no education, less than 5 years of schooling, 5–9 years of schooling and more than 10 years of schooling), Place of Residence (rural or urban), Living Arrangement (living alone, with spouse, with spouse and children, with children and others) and Number of Children Alive (no child, single child, 2 or more children). For socio-economic profile, Caste (scheduled caste, scheduled tribe, other backward caste and none), Religion (hindu, muslim and others), Monthly Per-capita Consumption Expenditure or MPCE Quintile (poorest, poor, middle, richer and richest), Economic Dependency (whether working or getting pension and dependent), Social Isolation (whether they meet or talk to their friends over phone), Level of Satisfaction in Current Living Arrangement (satisfied, neutral, not satisfied) etc. are controlled. Health behavior like consuming tobacco or alcohol which could be related to situation of mental and physical health along with Chronic Health Condition (suffering from chronic diseases or multimorbidity) are taken into consideration to understand the association of “caregiving factor” in identifying depression or poor self-rated health condition.

### Statistical analysis

Bivariate analysis shows prevalence of depressive symptoms and poor self-rated health along with socio-economic and demographic profile of caregivers and non-caregivers. To understand whether the prevalence is statistically significant or not, two sample proportion test was used [38]. As the outcome variable is binary in nature for both the health indicators, Pearson’s chi square test is performed for bivariate analysis. To analyse the association between different risk factors and depression or poor health, multivariable logistic regression is used within the caregiver’s sample and to compare the adjusted odd’s ratio with non-caregivers, interaction effect of caregiving is calculated in separate binary logistic regression models. The following logistic model is used for the analysis,

$$logit P= {b}_{0}+ {b}_{1}{X}_{1}+ {b}_{2}{X}_{2}+\dots + {b}_{k}{X}_{k}$$

Here, ${b}_{0}, {b}_{1}, {b}_{2}\dots ..{b}_{k}$ are coefficient of regression analysis, showed the relative effect of predictor variables on caregiver’s health.

To differentiate between the health condition of caregiver and non-caregiver under similar socio-economic condition, interaction terms have been introduced in logistic model.

$$logit P= {b}_{0}+ {b}_{1}{X}_{1}+ {b}_{2}{X}_{2}+{b}_{3}{X}_{1}{X}_{2}$$

Here, to understand interaction effect between ${X}_{1} and {X}_{2}$ the multiplicative term ${X}_{1}{X}_{2}$ has been introduced. Categories among explanatory variables are divided into two parts with interaction terms- caregivers and non-caregivers; e.g., for the variable Gender, there were primarily two categories ‘Male’ and ‘Female’, with using interaction terms we have created four categories i.e., ‘Male Caregiver’, ‘Male Non-Caregiver’, ‘Female Caregiver’ and ‘Female Non-Caregiver’. All the explanatory variables are modified using interaction terms and used in separate logistics regression models.

The general associative factors of depressive symptoms and poor health among adult population, identified through literature study, are controlled in all the regression models. To ensure absence of multicollinearity, variation inflation factor (VIF) test is done [39] for all the regression models and the mean values are always less than 4.5 (3.02–4.41), which indicated that the analysis does not suffer the effect of multicollinearity among the predictor variables. All the statistical analysis is done with the help of STATA 15 software.

## Results

Table 1 describes the study population according to their background characteristics. Each of the characteristics has been subdivided within two separate groups; caregivers and non-caregivers. 10.92% of the caregivers reported to possess poor self-rated health where as 29.30% of them have depressive symptoms.

**Table 1:** Characteristics of the study population stratified by caregivers and non-caregivers

Background Characteristics	Caregivers	Non- caregivers	*N* = 67,749
Self-Rated Heath			
Poor	10.92	10.61	6859
Good	89.08	89.39	60,890
Depression			
Depressed	29.30	27.58	17,874
Not depressed	70.70	72.42	49,875
Age Group			
< 45 years	14.42	8.41	6690
45–59 years	53.45	45.11	32,192
60–69 years	21.15	27.22	15,938
> 70 years	10.98	19.26	12,929
Sex			
Male	36.03	41.98	27,017
Female	63.97	58.02	40,732
Years of Schooling			
No Education	38.76	50.23	33,332
< 5 years	11.72	10.81	7076
5–9 years	22.33	21.17	14,362
> 10 years	27.19	17.80	12,979
Marital Status			
Currently Married	86.99	75.00	50,881
Widowed	9.69	22.28	14,576
Others	3.31	2.73	2292
MPCE Quintile			
Poorest	22.07	20.76	13,203
Poorer	20.12	21.37	13,574
Middle	17.29	20.47	13,550
Richer	17.58	19.74	13,696
Richest	22.93	17.66	13,726
Religion			
Hindu	77.30	82.29	48,285
Muslim	17.64	11.20	8552
Others	5.06	6.50	10,912
Caste			
Schedule Caste (SC)	17.69	19.82	11,935
Schedule Tribe (ST)	5.56	8.96	12,769
Other Backward Caste (OBC)	53.92	46.27	25,446
None	22.83	24.95	17,599
Residence			
Rural	62.97	68.98	45,825
Urban	37.03	31.02	21,924
Living Arrangement			
With Spouse	15.61	14.95	10,315
With Spouse and Children	70.51	59.11	39,663
With Children	8.08	18.40	12,646
Alone/ With Others	5.80	7.54	5125
Chronic Disease			
No Chronic Disease	53.26	55.28	35,300
Single Chronic Disease	26.17	26.90	19,473
Multimorbidity	20.57	17.82	12,976
Economic Dependency			
Dependent	43.93	51.76	35,479
Independent	56.07	48.24	32,270

*Note*: Individual level sampling weight is used

Table 2 and Table 3 shows the prevalence of depressive symptoms and self-reported poor health among adult caregivers and non-caregivers, respectively, and whether they are significantly different among each other along with their socio-economic and demographic characteristics. In most of the cases, prevalence of depressive symptoms is significantly higher among caregivers than non-caregivers. Among caregivers, 29% are diagnosed to have depressive symptoms, which is 27.58% for non-caregivers; and the difference is statistically significant (1.74; *p* < 0.05). On the other hand, 11% of the caregivers reported poor self-rated health (SRH). With increasing age there is a clear rising prevalence of depressive symptoms; for the age group 60–69 years the difference in depressive symptom is maximum between two groups (7.62, *p* < 0.05). According to marital status, widows have maximum difference between caregivers and non-caregivers (11.7, *p* < 0.05) in depressive symptom but for self-rated health the difference is way lower (4.45, *p* < 0.05). Rural caregivers have higher prevalence for both depression (4.96, *p* < 0.05) and SRH (1.15, *p* < 0.05) than their counterpart whereas in urban areas caregivers are less depressed (-3.4, *p* < 0.05) and reports poor SRH a little less. Living with spouse not children or living with children without spouse creates similar amount of difference in depression between caregivers and non-caregivers (10.53 & 12.76, *p* < 0.05). Along with type of living arrangement, not being satisfied with the current living arrangement shows significant maximum difference between caregivers and non-caregivers (12.81, *p* < 0.05) in prevalence of depressive symptoms. Though being socially isolated doesn’t create significant difference in depression among caregivers and non-caregivers but caregivers who are not socially isolated are more depressed (5.29, *p* < 0.05).

**Table 2:** Prevalence of depressive symptoms among caregivers and non-caregivers in India, LASI Wave 1, 2017–18

Background Characteristics	Caregivers		Non-Caregivers		Difference	Proportion Test (*p* value)
	Prevalence (*N* = 2522)	χ^2^	Prevalence (*N* = 65,227)	χ^2^		
Age Group		χ^2^ = 16.99 *p* = 0.001		χ^2^ = 334.86 *p* = 0.000		
<45 years	15.94		21.91		-5.97	0.505
45–59 years	28.43		26.19		2.24	0.000
60–69 years	36.21		28.59		7.62	0.000
> 70 years	38.43		32.05		6.38	0.000
Sex		χ^2^ = 13.99 *p* = 0.000		χ^2^ = 72.03 *p* = 0.000		
Male	26.58		25.6		0.98	0.069
Female	30.86		29.00		1.86	0.000
Years of Schooling		χ^2^ = 52.40 *p* = 0.000		χ^2^ = 569.39 *p* = 0.000		
No Education	37.27		31.48		5.79	0.000
< 5 years	30.74		27.72		3.02	0.186
5–9 years	24.67		24.35		0.32	0.256
> 10 years	21.25		20.45		0.8	0.003
Marital Status		χ^2^ = 32.76 *p* = 0.000		χ^2^ = 583.43 *p* = 0.000		
Currently Married	26.87		25.16		1.71	0.000
Widowed	47.10		35.40		11.7	0.000
Others	43.07		31.31		11.76	0.027
MPCE Quintile		χ^2^ = 11.36 *p* = 0.023		χ^2^ = 92.63 *p* = 0.000		
Poorest	35.09		29.85		5.24	0.000
Poorer	23.20		27.15		-3.95	0.474
Middle	36.19		28.46		7.73	0.000
Richer	25.25		25.46		-0.21	0.047
Richest	27.12		26.79		0.33	0.003
Religion		χ^2^ = 2.61 *p* = 0.271		χ^2^ = 259.62 *p* = 0.000		
Hindu	31.42		27.68		3.74	0.000
Muslim	18.69		30.21		-11.52	0.181
Others	34.26		21.82		12.44	0.000
Caste		χ^2^ = 17.95 *p* = 0.000		χ^2^ = 251.07 *p* = 0.000		
Schedule Caste (SC)	37.99		30.88		7.11	0.000
Schedule Tribe (ST)	22.47		25.31		-2.84	0.107
Other Backward Caste (OBC)	27.74		27.94		-0.2	0.001
None	27.37		24.76		2.61	0.020
Residence		χ^2^ = 5.17 *p* = 0.023		χ^2^ = 59.13 *p* = 0.000		
Rural	33.2		28.24		4.96	0.000
Urban	22.72		26.12		-3.4	0.004
Living Arrangement		χ^2^ = 43.03 *p* = 0.000		χ^2^ = 642.40 *p* = 0.000		
With Spouse	38.55		28.02		10.53	0.000
With Spouse and Children	24.2		24.43		-0.23	0.000
With Children	45.42		32.66		12.76	0.000
With Others	45.57		39.54		6.03	0.391
Chronic Disease		χ^2^ = 15.55 *p* = 0.000		χ^2^ = 349.73 *p* = 0.000		
No Chronic Disease	26.77		24.97		1.8	0.000
Single Chronic Disease	36.11		28.40		7.71	0.000
Multimorbidity	27.33		34.55		-7.22	0.016
Social Isolation		χ^2^ = 15.63 p = 0.000		χ^2^ = 336.05 p = 0.000		
Isolated	29.35		29.12		0.23	0.000
Not Isolated	29.27		23.98		5.29	0.000
Economic Dependency		χ^2^ = 11.22 *p* = 0.001		χ^2^ = 357.10 *p* = 0.000		
Dependent	32.73		30.74		1.99	0.000
Independent	26.69		24.25		2.44	0.000
Satisfaction in Current Living Arrangement		χ^2^ = 171.45 *p* = 0.000		χ^2^ = 2400 *p* = 0.000		
Satisfied	22.63		23.05		-0.42	0.002
Neutral	44.39		40.71		3.68	0.001
Not Satisfied	65.85		53.04		12.81	0.010
**Overall**	**29.32**		**27.58**		**1.74**	**0.000**

*Note*: Individual level sampling weight is used

**Table 3:** Prevalence of poor self rated health among caregivers and non-caregivers in India, LASI Wave 1, 2017–18

Background Characteristics	Caregivers		Non-Caregivers		Difference	Proportion Test (*p* value)
	Prevalence (*N* = 2522)	χ^2^	Prevalence (*N* = 65,227)	χ^2^		
Age Group		χ^2^ = 39.16 *p* = 0.000		χ^2^ = 1600.00 *p* = 0.000		
< 45 years	8.16		5.41		2.75	0.927
45–59 years	8.68		7.13		1.55	0.003
60–69 years	13.65		12.30		1.35	0.574
> 70 years	20.62		18.95		1.67	0.973
Sex		χ^2^ = 0.01 *p* = 0.920		χ^2^ = 16.02 *p* = 0.000		
Male	11.72		10.39		1.33	0.019
Female	10.48		10.78		-0.3	0.003
Years of Schooling		χ^2^ = 16.23 *p* = 0.001		χ^2^ = 279.34 *p* = 0.000		
No Education	15.10		12.36		2.74	0.319
< 5 years	11.22		12.54		-1.32	0.382
5–9 years	10.37		9.43		0.94	0.429
> 10 years	5.32		6.01		-0.69	0.440
Marital Status		χ2 = 18.78 *p* = 0.000		χ^2^ = 462.06 *p* = 0.000		
Currently Married	10.18		9.33		0.85	0.163
Widowed	19.67		15.22		4.45	0.019
Others	5.30		8.95		-3.65	0.381
MPCE Quintile		χ^2^ = 3.48 *p* = 0.481		χ^2^ = 18.67 *p* = 0.001		
Poorest	10.98		10.41		0.57	0.003
Poorer	9.51		10.39		-0.88	0.065
Middle	9.27		10.91		-1.64	0.373
Richer	10.84		10.23		0.61	0.211
Richest	8.77		11.23		-2.46	0.993
Religion		χ^2^ = 0.53 *p* = 0.766		χ^2^ = 167.29 *p* = 0.000		
Hindu	10.74		10.18		0.56	0.071
Muslim	10.03		13.49		-3.46	0.366
Others	16.77		11.25		5.52	0.101
Caste		χ^2^ = 15.59 *p* = 0.001		χ^2^ = 206.68 *p* = 0.000		
Schedule Caste (SC)	16.54		12.35		4.19	0.049
Schedule Tribe (ST)	7.87		7.97		-0.1	0.401
Other Backward Caste (OBC)	9.34		9.99		-0.65	0.245
None	9.70		10.69		-0.99	0.118
Residence		χ^2^ = 1.87 *p* = 0.171		χ^2^ = 82.22 *p* = 0.000		
Rural	12.76		11.61		1.15	0.030
Urban	7.80		8.42		-0.62	0.005
Living Arrangement		χ^2^ = 14.85 *p* = 0.002		χ^2^ = 439.82 *p* = 0.000		
With Spouse	11.13		11.48		-0.35	0.070
With Spouse and Children	9.94		8.77		1.17	0.167
With Children	19.68		13.83		5.85	0.129
With Others	10.50		15.82		-5.32	0.128
Social Isolation		χ^2^ = 16.68 *p* = 0.000		χ^2^ = 337.79 *p* = 0.000		
Isolated	12.54		11.70		0.84	0.248
Not Isolated	8.37		8.04		0.33	0.057
Economic Dependency		χ^2^ = 29.50 *p* = 0.000		χ^2^ = 1100.00 *p* = 0.000		
Dependent	15.52		14.53		0.99	0.849
Independent	7.38		6.50		0.88	0.036
Satisfaction in Current Living Arrangement		χ^2^ = 106.95 *p* = 0.000		χ^2^ = 1400.00 *p* = 0.000		
Satisfied	7.25		8.80		-1.55	0.356
Neutral	16.45		13.27		3.18	0.943
Not Satisfied	36.07		30.71		5.36	0.060
**Overall**	**10.92**		**10.62**		**0.3**	**0.555**

*Note*: Individual level sampling weight is used

**Fig. 1:**
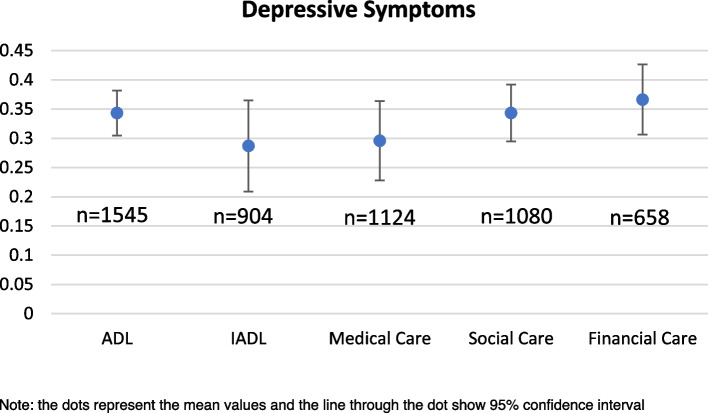
Prevalence of depressive symptoms among the caregivers according to types of care they provide

**Fig. 2:**
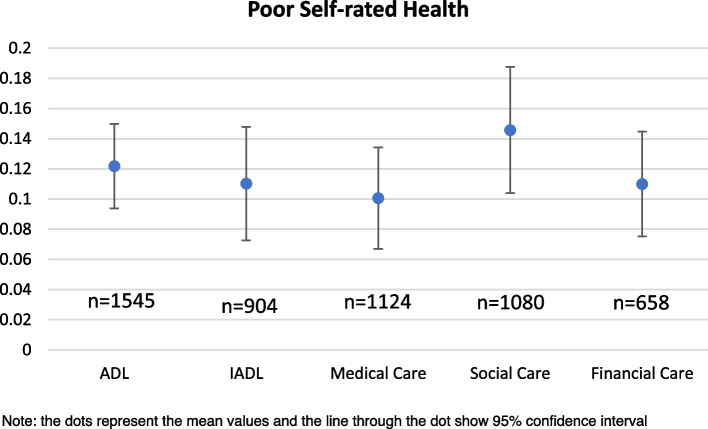
Prevalence of poor self-rated health among the caregivers according to types of care they provide

**Fig. 3:**
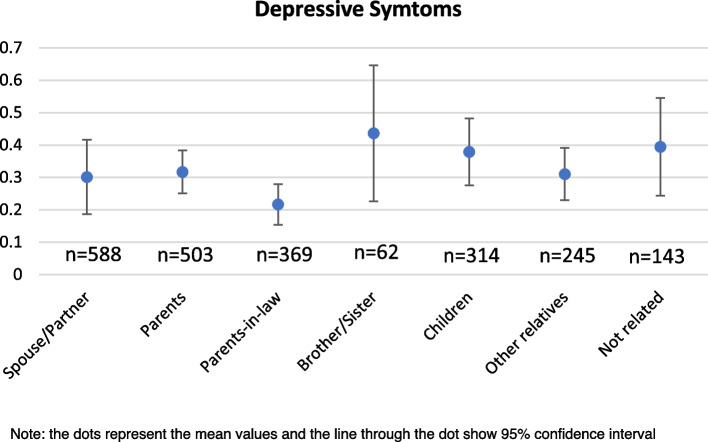
Prevalence of depressive symptoms among the caregivers according to the relationship with care receivers

**Fig. 4:**
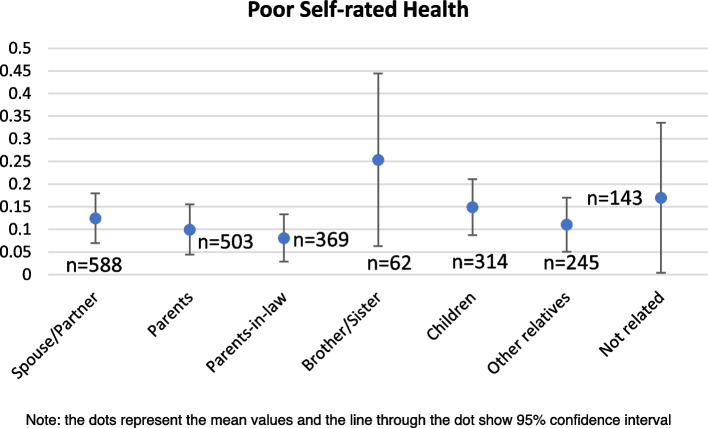
Prevalence of poor self-rated health among the caregivers according to the relationship with care receivers

The variation in prevalence of depressive symptoms and poor SRH among caregivers with the type of care provided and the relationship with the care recipient is explained in the Figs. 1,2,3,4 respectively. In Figs. 1 and 2, the pattern of prevalence of depression and poor SRH shows similarity in all the types of care provided except the financial support. In case of financial care, the prevalence of depressive symptoms is highest (36%) among the caregivers whereas the prevalence of poor SRH is 11% among them. Caregivers providing ADL care and supporting emotionally and socially have similar prevalence of depressive symptoms (34% in both the cases) and 29% among those who provided IADL and medical care reported to have depressive symptoms. In case of poor SRH, caregivers providing emotional or social support in any of the family member or non-family member have highest prevalence of 15%. In Figs. 3 and 4, 44% of the caregivers providing care to their brothers and sisters have depressive symptoms which is around 25% in case of prevalence of poor SRH. Among the other relationships, 22% and 8% of the caregivers who provided care to their parents-in-law reported to have depressive symptoms and poor SRH respectively; which is lowest compared to other relationships.

Table 4. depicts the association between the explanatory variables with depressive symptoms and poor SRH among the caregivers in different socio-economic settings. Time spent in caregiving significantly increases the probability of being depressed (AOR 1.59, CI 1.16–2.18) as well as reporting poor SRH (AOR 1.73, CI 1.11–2.69). With increasing years of schooling, the probability of being depressed among the caregivers, decreases at a constant rate. Caregivers, who are not satisfied with their current living arrangement, reported manifold increased probability of having depression (AOR 4.22, CI 2.93–6.08) and poor SRH (AOR 4.42, CI 2.88–6.78). In providing care for ADL and financial support, caregivers reported to have 1.36 times higher odds of being depressed than those who does not provide those supports, which is opposite in case of medical care (AOR 0.68, CI 0.54–0.86). Providing care to parents-in-law seems to affect least in case of depression than any other relationships with the care recipient. Providing care to own parents, partner/spouse and children increases the chance of having depressive symptoms by 1.66, 1.80 and 1.50 times respectively. Economically dependent caregivers reported to have 1.17 times increased probability of poor SRH.

**Table 4:** Association between background characteristics of caregivers with depressive symptoms and poor self-rated health among adults in India, LASI Wave 1, 2017–18

Explanatory Variables	Depressive Symptoms		Poor Self-rated Health	
	AOR (95% CI)	*p*-Value	AOR (95% CI)	*p*-Value
Hours Spent for Caregiving				
Less than 40 h/week ®				
More than 40 h/week	1.59 (1.16, 2.18)	0.004	1.73 (1.11, 2.69)	0.016
Age Group				
< 45 years ®				
45–59 years	1.38 (0.96, 1.99)	0.079	1.52 (0.80, 2.87)	0.2
60–69 years	1.48 (0.97, 2.25)	0.069	1.94 (0.96, 3.92)	0.064
> 70 years	1.13 (0.69, 1.85)	0.616	2.96 (1.39, 6.28)	0.005
Sex				
Male ®				
Female	1.26 (0.95, 1.67)	0.109	0.97 (0.63, 1.50)	0.899
Education				
No Education ®				
Less than 5 years	0.72 (0.51, 1.01)	0.055	1.48 (0.92, 2.38)	0.108
5–9 years	0.70 (0.53, 0.94)	0.016	0.95 (0.60, 1.49)	0.813
More than 10 years	0.70 (0.50, 0.98)	0.038	0.91 (0.53, 1.55)	0.718
Marital Status				
Currently Married ®				
Widowed	1.07 (0.39, 2.95)	0.9	1.27 (0.26, 6.17)	0.765
Others	1.65 (0.55, 5.00)	0.375	0.41 (0.06, 2.85)	0.367
MPCE Quintile				
Poorest ®				
Poorer	0.73 (0.52, 1.03)	0.073	0.95 (0.55, 1.64)	0.859
Middle	1.14 (0.81, 1.59)	0.451	1.07 (0.62, 1.85)	0.797
Richer	0.78 (0.55, 1.10)	0.153	1.24 (0.73, 2.11)	0.428
Richest	0.94 (0.67, 1.32)	0.736	1.62 (0.97, 2.71)	0.063
Religion				
Hindu ®				
Muslim	0.95 (0.68, 1.32)	0.757	1.43 (0.88, 2.32)	0.154
Others	0.65 (0.44, 0.95)	0.026	1.21 (0.69, 2.12)	0.502
Multimorbidity				
No Chronic Disease ®				
Single Chronic Disease	1.24 (0.97, 1.58)	0.087	…	…
Multimorbidity	1.50 (1.14, 1.99)	0.004	…	…
Caste				
None ®				
Scheduled Caste (SC)	1.22 (0.88, 1.68)	0.229	1.58 (0.98, 2.55)	0.059
Scheduled Tribe (ST)	0.60 (0.40, 0.91)	0.015	0.49 (0.25, 0.97)	0.041
Other Backward Class (OBC)	1.06 (0.82, 1.37)	0.642	1.14 (0.77, 1.71)	0.507
Residence				
Urban ®				
Rural	1.14 (0.90, 1.45)	0.287	1.20 (0.82, 1.74)	0.354
Living Arrangement				
Living with Others ®				
Living with Spouse	1.39 (0.46, 4.15)	0.559	1.15 (0.21, 6.26)	0.872
Living with Spouse and Children	1.15 (0.39, 3.35)	0.803	1.21 (0.23, 6.35)	0.822
Living with Children	1.70 (0.92, 3.15)	0.09	1.46 (0.59, 3.61)	0.415
Satisfaction in Current Living Arrangement				
Satisfied ®				
Neutral	2.23 (1.69, 2.93)	0.000	1.47 (0.95, 2.26)	0.084
Not Satisfied	4.22 (2.93, 6.08)	0.000	4.42 (2.88, 6.78)	0.000
Consume Tobacco				
No ®				
Yes	0.77 (0.60, 0.99)	0.043	1.04 (0.71, 1.51)	0.842
Consume alcohol				
No ®				
Yes	1.36 (0.93, 1.98)	0.117	1.57 (0.92, 2.68)	0.101
Social Isolation				
No ®				
Yes	1.08 (0.87, 1.36)	0.473	1.57 (1.10, 2.24)	0.013
Types of Care Provided				
ADL Care				
No ®				
Yes	1.36 (1.07, 1.73)	0.012	1.10 (0.76, 1.60)	0.609
IADL Care				
No ®				
Yes	1.18 (0.94, 1.47)	0.149	1.26 (0.90, 1.77)	0.176
Social Care				
No ®				
Yes	1.01 (0.80, 1.27)	0.925	0.81 (0.56, 1.17)	0.244
Medical Care				
No ®				
Yes	0.68 (0.54, 0.86)	0.001	1.29 (0.89, 1.80)	0.165
Financial Care				
No ®				
Yes	1.35 (1.04, 1.76)	0.024	0.87 (0.57, 1.32)	0.516
Care Recipient				
Parent-in-law ®				
Spouse/Partner	1.80 (1.25, 2.59)	0.002	1.32 (0.74, 2.35)	0.353
Parents	1.66 (1.14, 2.42)	0.008	1.03 (0.55, 1.90)	0.937
Brothers/Sisters	1.52 (0.75, 3.06)	0.244	1.24 (0.44, 3.51)	0.679
Children	1.50 (1.01, 2.24)	0.045	1.23 (0.66, 2.30)	0.518
Other Relatives	1.61 (1.05, 2.46)	0.029	1.15 (0.58, 2.28)	0.694
Not Related	1.80 (1.08, 3.01)	0.024	1.38 (0.62, 3.07)	0.434
Economic Dependency				
Independent ®				
Dependent	1.17 (0.92, 1.47)	0.201	1.72 (1.19, 2.49)	0.004
Constant	0.10 (0.03, 0.35)		0.01 (0.00, 0.10)	
No. of Observations	2522			

*Note*: *AOR* Adjusted Odds Ratio, *CI* Confidence Interval, ® Reference Category, *ADL* Activities in Daily Living, *IADL* Instrumental Activities in Daily Living

The interaction effect of caregiving on depression and poor SRH is depicted in Table 5 where each of the category is stratified into caregiver and non-caregiver group and the non-caregivers are kept as reference category. In all of the socio-economic strata, caregivers show higher chance of being depressed and possessing poor health than non-caregivers. The odds of being depressed increases at a constant rate with increasing age of the caregivers; for the age group 45–59 (AOR 1.42, CI 1.11–1.82) and 60–69 (AOR 1.41, CI 1.10–1.81-); whereas the likelihood of having poor SRH increases up to 1.86 times (CI 1.31–2.65) for the caregivers than the non-caregivers belonging to 45–59 years of age. The risk of depression is higher for widowed caregivers (AOR 1.58, CI 1.12–2.23) which is 1.36 times higher for married caregiver in comparison to non-caregivers of same marital status. And similarly, odds of having poor SRH (AOR 1.90, CI 1.19–3.02) is almost twice for the widows who provide care. In urban and rural areas, probability of having depression and poor SRH vary significantly; putting rural caregivers at a little higher risk for depression (AOR 1.42, CI 1.21–1.81) than rural non-caregivers; though odds of having poor SRH does not vary much according to place of residence (AOR 1.60 for urban and AOR 1.58 for rural) of the caregivers. The gender differential has an interesting pattern when compared with non-caregivers; Male caregivers shows 1.26 times higher odds of having depressive symptoms than male non-caregivers; whereas the odds increase up to 1.51 times for female caregivers. But male caregivers report 1.72 times higher odds of reporting poor SRH than male non-caregivers which is only 1.48 times for females. Caregivers, who never attended school, reported to have higher odds of having depressive symptoms (AOR 1.59 CI 1.23–2.04).

**Table 5:** Regression analysis with interaction effect of caregiving on mental and self-rated health among adults in India, LASI Wave 1, 2017–18

Background Characteristics	Depressive Symptoms		Poor Self-Rated Health	
	AOR (95% CI)	*p*-Value	AOR (95% CI)	*p*-Value
Caregiver				
No®				
Yes	1.28 (1.17,1.41)	0.000	1.11 (1.01,1.23)	0.009
Age Group with Interaction				
< 45 years * Non-Caregiver ®				
< 45 years * Caregiver	1.09 (0.77, 1.55)	0.632	1.33 (0.71, 2.49)	0.382
45–59 years * Non-Caregiver®				
45–59 years * Caregiver	1.42 (1.11, 1.82)	0.005	1.86 (1.31, 2.65)	0.001
60–69 years * Non-Caregiver®				
60–69 years * Caregiver	1.41 (1.10, 1.81)	0.007	1.35 (0.91, 2.01)	0.137
> 70 years * Non-Caregiver®				
> 70 years * Caregiver	1.21 (0.87, 1.70)	0.256	1.45 (0.94, 2.23)	0.091
Marital Status with Interaction				
Currently Married * Non-Caregiver ®				
Currently Married * Caregiver	1.36 (1.08, 1.72)	0.009	1.55 (1.12, 2.15)	0.009
Widowed * Non-Caregiver®				
Widowed * Caregiver	1.58 (1.12, 2.23)	0.009	1.90 (1.19, 3.02)	0.009
Others * Non-Caregiver®				
Other * Caregiver	1.84 (1.11, 3.04)	0.018	1.03 (0.38, 2.78)	0.954
Residence with Interaction				
Urban * Non-Caregiver ®				
Urban * Caregiver	1.34 (1.04, 1.74)	0.025	1.60 (1.10, 2.33)	0.015
Rural * Non-Caregiver®				
Rural * Caregiver	1.42 (1.12, 1.81)	0.004	1.58 (1.21, 2.22)	0.009
Sex with Interaction				
Female * Non-Caregiver ®				
Female * Caregiver	1.51 (1.18, 1.93)	0.001	1.48 (1.04, 2.10)	0.029
Male * Non-Caregiver®				
Male * Caregiver	1.26 (0.98, 1.63)	0.070	1.72 (1.20, 2.45)	0.003
Literate with Interaction				
Literate * Non-Caregiver ®				
Literate * Caregiver	1.20 (0.94, 1.54)	0.143	1.52 (1.08, 2.16)	0.018
Non-Literate * Non-Caregiver®				
Non-Literate * Caregiver	1.59 (1.23, 2.04)	0.000	1.64 (1.15, 2.35)	0.006
MPCE Quintile with Interaction				
Poorest * Non-Caregiver ®				
Poorest * Caregiver	1.53 (1.13, 2.07)	0.006	1.81 (1.15, 2.83)	0.010
Poorer * Non-Caregiver®				
Poorer * Caregiver	1.03 (0.76, 1.40)	0.846	1.42 (0.91, 2.33)	0.123
Middle * Non-Caregiver®				
Middle * Caregiver	1.67 (1.25, 2.22)	0.000	1.34 (0.86, 2.07)	0.197
Richer * Non-Caregiver®				
Richer * Caregiver	1.27 (0.95, 1.71)	0.105	1.82 (1.21, 2.73)	0.000
Richest * Non-Caregiver®				
Richest * Caregiver	1.41 (1.05, 1.87)	0.022	1.60 (1.06, 2.42)	0.025
Living Arrangement with Interaction				
With Others * Non-Caregiver ®				
With Others * Caregiver	1.36 (0.85, 2.08)	0.174	1.27 (0.63, 2.55)	0.503
With Spouse * Non-Caregiver ®				
With Spouse * Caregiver	1.57 (1.15, 2.15)	0.004	1.43 (0.86, 2.42)	0.169
With Spouse & Children * Non-Caregiver ®				
With Spouse & Children * Caregiver	1.33 (1.05, 1.69)	0.018	1.57 (1.12, 2.20)	0.008
With Children * Non-Caregiver ®				
With Children * Caregiver	1.86 (1.31, 2.62)	0.000	2.02 (1.26, 3.24)	0.003
Social Isolation with Interaction				
Isolated * Non-Caregiver ®				
Isolated * Caregiver	1.40 (1.10, 1.79)	0.007	1.61 (1.14, 2.26)	0.006
Not Isolated * Non-Caregiver ®				
Not Isolated * Caregiver	1.43 (1.11, 1.84)	0.006	1.54 (1.06, 2.24)	0.024
Economic Dependency with Interaction				
Independent * Non-Caregiver ®				
Independent * Caregiver	1.42 (1.1, 1.81)	0.005	1.75 (1.23, 2.50)	0.002
Dependent * Non-Caregiver ®				
Dependent * Caregiver	1.40 (1.09, 1.81)	0.009	1.46 (1.03, 2.07)	0.034
No. of Observations	67,749			

*Note*: *AOR* Adjusted Odds Ratio, *CI* Confidence Interval, *®* Reference Category

## Discussion

This research is focused on health condition of informal caregivers; both in terms of mental health and overall health satisfaction. With help of the CESD scale depressive symptoms are identified. On the other hand, self-rated health is as representative of overall health condition and satisfaction from the point of view of the respondent himself. The study has explained the association of role of caregiving with depressive symptoms and poor self-rated health by comparing caregivers with non-caregivers of different socio-economic strata. Probable other associated factors of depression and poor physical health in later life, identified through literature, are controlled in all the models to identify whether caregiving role induces poor mental and physical health condition. The study proves that “caregiving factor”, irrespective of all socio-economic characteristics, has association in increasing the likelihood of possessing depressive symptoms and poor SRH significantly in almost all socio-economic strata. The major conclusion of this study aligns in similar direction with the study by Aarti et al., 2019, [40] which shows family caregivers suffer from moderate-severe depression as well as leads poor quality of life.

Among different types of care, 68% of the caregivers are engaged in providing care for ADL, which requires enduring intensive physical and emotional responsibility almost all day long. Almost half of the caregivers (49%), who provided care for more than 40 h a week, are reported to have depressive symptoms. Providing care for 40 h per week is equivalent to a full-time occupation but here is rendered in addition to any other job or responsibilities the caregiver may have [41, 42]. Along with care in ADL, providing financial support increases the odds of being depressed by 1.4 times. But the exact opposite situation is for those who provided medical support, such as accompanying to hospital or changing bandages and taking care of medications, where the risk of depression is 33% less. Possible financial strain caused due to health expenditure may be a probable factor of increasing depressive symptoms among those who provide financial support [43, 44]. Similarly, economic dependency of caregivers show higher risk of being depressed. On the other hand, accompanying to the hospital in times of need or managing the medication is not such a stressful liability for most of the cases. And may, in fact, provide some emotional satisfaction, resulting in reduced odds of being depressed.

Several studies identified marital status as having a direct relationship with depression among adult population; where widowed people are the most vulnerable [45]. The result of this study is consistent with previous literatures on this point, 3 among 5 widowed caregivers reported to have depressive symptoms. On the contrary, among married couples, spousal caregiving shows highest probability of being depressed with restrictions in activity with any one of the partners, which is consistent with previous research on this phenomenon in developed countries [46–48], whereas providing care to parent-in-law shows lowest probability of having depression. Along with other East Asian Countries, in Indian social structure also, it is highly accepted that daughters-in-law are supposed to be the primary caregivers to the parents-in-law in a household and thus in this filial piety the caregiving is accepted as a duty but not an unpaid care service which leads to lower likelihood of having depression among caregivers [49–52]. Gender differentials in health outcome corresponding to caregiving is reflected in the interaction effect and female caregivers are more susceptible to depressive symptoms than male caregivers. But in case of poor self-rated health male caregivers reported to have 1.72 times higher odds of having poor health, which is 1.48 times higher for the female caregivers. This signifies the aforesaid cultural setting of gender biased role of caregiving [53, 54].

Co-residence plays an important role in caregiving process as well as in developing depressive symptoms. Caregiving significantly increases the risk of depression and poor SRH for the caregiver living only with children and others, with spouse being absent due to their death or separation or divorce. Here, absence of spouse leads to conflicting role of the caregivers with other relationships and makes the job of caregiving burdensome [55]. Similarly, Caregivers, not satisfied with their current residential arrangement, have 4 times increased risk of having depressive symptoms and poor self-rated health. The finding is consistent with previous researches [56]; in Indian emigrant households, where the wives left behind to live separate from their spouse, feel the duty of caregiving burdensome [33]. Even in contemporary Indian rural residence, a joint family is most common family set up, having the elderly family members co-reside with the younger generation. With increasing family members, the burden of providing care also increases which in turn increases the risk of being depressed and reporting poor health condition. On the contrary, in urban areas the joint family culture is not that prevalent. Availability of paid formal caregivers also lowers the burden of caregiving for a family member in urban areas. Existing health related problems among the caregivers only aggravate the likelihood of depression; caregivers having multimorbidity are more likely to have depressive symptoms than the caregivers with no chronic disease and the non-caregivers with multimorbidity [3].

In contrast to existing literature on caregiving in Indian context, which tend to focus on a particular community setting or are based on area specific small-scale survey, this study uses the nationally representative sample survey to provide a wholesome picture of health condition of informal caregivers. But the research is not completely free from limitations. Whether caregiving role is responsible for onsetting the depressive symptoms or poor health outcome, could not be analysed with only cross-sectional data. With growing awareness about the cause of poor mental health conditions of all age groups, identifying the hidden or endogenous causes are important policy concern. For ease of analysis the health outcome is made binary, a more elaborated outcome scale could have provided more insightful understanding of how exactly the health is changing and very minor differences could have been captured. Due to limitation of data, comparative analysis among the caregivers, according to the type of disease of the care recipient could not be done in this research. However, disease specific burden of caregiving and therapeutic solution to adjustment of the caregiver, specifically in Indian context, is a clinical research concern and can be fulfilled by further research. State-wise detailed analysis can also bring variations in cultural and social scenario as different states in India are in different pace of demographic transition and the burden of age cumulative dependency is not similar for all the states. But it is evident that, in near future, India has to be prepared to provide age friendly living conditions to its growing greying population and the need for care has to be met at a household level, not through institutionalization. Thus, along with the health condition of the ageing population, the health of the adult population, providing care to the elderly, needs to be focused on to ensure a healthy co-residence.

## Conclusions

This empirical study focused on the effect of caregiving on caregivers’ health, in terms of depression and self-rated health. Using CES-D scale, the prevalence of depressive symptoms is estimated- within the caregivers and simultaneously a comparison between caregivers and non-caregivers is made, taking into consideration different socio-economic and demographic characteristics. The study observes that, in every background characteristic, caregiving does increase the risk of having depressive symptoms along with poor self-rated health. Caregivers aged between 45–69 years, widowed, not satisfied with their current living condition, having multimorbidity and those who are economically dependent are more depressed than non-caregivers of same social- economic status. Caregivers, who are providing care for more than 40 h a week and spousal caregiving are reported to have higher risk of depression and poor self-rated health. In human life cycle, in the early stage of life, caregiving is one directional; from parent to children, but in advance stage of life caregiving advances from a bi-directional to a multidirectional process; where a person simultaneously become a caregiver and care receiver as well. Thus, it requires immense psychological as well as physiological strength and involvement to be a primary caregiver. To cope up and adjust with the process of caregiving the health of the caregiver, specially, psychological health, needs more attention and acknowledgement.

## Supplementary Information

**Additional file 1:**

## Data Availability

The data of LASI is freely available in public domain through https://g2aging.org/.

## Abbreviations

ADL: Activities in Daily Living
IADL: Instrumental Activities in Daily Living
CES-D: Centre for Epidemiologic Studies Depression
SRH: Self-rated Health
AOR: Adjusted Odds Ratio
CI: Confidence Interval
SC: Scheduled Caste
ST: Scheduled Tribe
OBC: Other Backward Caste

## References

[1] Prince MJ, Wu F, Guo Y, Robledo LM, O'Donnell M, Sullivan R, Yusuf S (2015). The burden of disease in older people and implications for health policy and practice. The Lancet.

[2] Ugargol AP, Hutter I, James KS, Bailey A. Caregiving patterns to older adults in India. In Extended Abstract, PAA meetings, San Diego 2015. https://paa2015.princeton.edu/papers/153320

[3] Ajay S, Kasthuri A, Kiran P, Malhotra R (2017). Association of impairments of older persons with caregiver burden among family caregivers: findings from rural South India. Arch Gerontol Geriatr.

[4] Sebastian SI, Sekhar TV. Intergenerational care and support for elderly: evidence from Kerala State, India. In European Population Conference 2012 (pp. 1–17). https://epc2012.princeton.edu/abstracts/120232

[5] Maresova P, Lee S, Fadeyi OO, Kuca K (2020). The social and economic burden on family caregivers for older adults in the Czech Republic. BMC Geriatr.

[6] Giebel CM, Davies S, Clarkson P, Sutcliffe C, Challis D, HoSt-D (Home Support in Dementia) Programme Management Group. Costs of formal and informal care at home for people with dementia: ‘Expert panel’ opinions from staff and informal carers. Dementia. 2019; doi:10.1177/1471301216665705 10.1177/1471301216665705 27554789

[7] Joo H, Wang G, Yee SL, Zhang P, Sleet D (2017). Economic burden of informal caregiving associated with history of stroke and falls among older adults in the US. Am J Prev Med.

[8] Rote SM, Moon HE, Kacmar AM, Moore S (2022). Exploring coping strategies and barriers in dementia care: a mixed-methods study of African American family caregivers in Kentucky. J Appl Gerontol.

[9] Graessel E, Berth H, Lichte T, Grau H (2014). Subjective caregiver burden: validity of the 10-item short version of the burden scale for family caregivers BSFC-s. BMC Geriatr.

[10] Kleinman A (2010). Caregiving: Its role in medicine and society in America and China. Ageing Int.

[11] Moss KO, Kurzawa C, Daly B, Prince-Paul M (2019). Identifying and addressing family caregiver anxiety. JHPN.

[12] Or R, Kartal A (2019). Influence of caregiver burden on well-being of family member caregivers of older adults. Psychogeriatr.

[13] Hoffman GJ, Wallace SP (2018). The cost of caring: Economic vulnerability, serious emotional distress, and poor health behaviors among paid and unpaid family and friend caregivers. Res Aging.

[14] Schulz R, Sherwood PR (2008). Physical and mental health effects of family caregiving. J Soc Work Educ.

[15] Schulz R, Visintainer P, Williamson GM (1990). Psychiatric and physical morbidity effects of caregiving. J Gerontol.

[16] Pinquart M, Sörensen S (2003). Differences between caregivers and noncaregivers in psychological health and physical health: a meta-analysis. Psychol Aging.

[17] Schulz R, Beach SR (1999). Caregiving as a risk factor for mortality: the caregiver health effects study. JAMA.

[18] Gupta R, Pillai VK, Levy EF (2012). Relationship quality and elder caregiver burden in India. J Soc Interven: Theo Prac.

[19] Gupta R, Rowe N, Pillai VK (2009). Perceived caregiver burden in India: implications for social services. Affilia.

[20] Cuijpers P (2005). Depressive disorders in caregivers of dementia patients: a systematic review. Aging Mental Health.

[21] Von Känel R, Mausbach BT, Dimsdale JE, Ziegler MG, Mills PJ, Allison MA, Patterson TL, Ancoli-Israel S, Grant I (2019). Refining caregiver vulnerability for clinical practice: determinants of self-rated health in spousal dementia caregivers. BMC Geriatr.

[22] Lambert SD, Bowe SJ, Livingston PM, Heckel L, Cook S, Kowal P, Orellana L (2017). Impact of informal caregiving on older adults’ physical and mental health in low-income and middle-income countries: a cross-sectional, secondary analysis based on the WHO’s Study on global AGEing and adult health (SAGE). BMJ Open.

[23] Abdollahpour I, Nedjat S, Noroozian M, Salimi Y, Majdzadeh R (2014). Caregiver burden: the strongest predictor of self-rated health in caregivers of patients with dementia. J Geriatr Psychiatr Neuro.

[24] Pinquart M, Sörensen S. Correlates of physical health of informal caregivers: a meta-analysis. J. Gerontol. Series B: Psychol. Sc. Soc. Sc. 2007; doi: 10.1093/geronb/62.2.p126 10.1093/geronb/62.2.p126 17379673

[25] Brown SL (2007). Health effects of caregiving: studies of helping behavior needed!. Alzheimer's Care Today.

[26] Tarlow BJ, Wisniewski SR, Belle SH, Rubert M, Ory MG, Gallagher-Thompson D (2004). Positive aspects of caregiving: Contributions of the REACH project to the development of new measures for Alzheimer’s caregiving. Res Aging.

[27] Roth DL, Dilworth-Anderson P, Huang J, Gross AL, Gitlin LN (2015). Positive aspects of family caregiving for dementia: differential item functioning by race. J Gerontol B Psychol Sci Soc Sci.

[28] Roth DL, Haley WE, Hovater M, Perkins M, Wadley VG, Judd S (2013). Family caregiving and all-cause mortality: findings from a population-based propensity-matched analysis. Am J Epidemiol.

[29] Chan CK, Vickers T, Barnard A (2020). Meaning through caregiving: a qualitative study of the experiences of informal carers. Br J Soc Work.

[30] Ory MG, Hoffman RR, Yee JL, Tennstedt S, Schulz R (1999). Prevalence and impact of caregiving: a detailed comparison between dementia and nondementia caregivers. Gerontol.

[31] Kumari A, Sekher TV. Elderly as Family Caregivers: Burden and Challenges in India. In Gerontological Concerns and Responses in India 2021 (pp. 159–181). Springer, Singapore.

[32] Kumar R, Saini R (2013). Extent of burden and coping strategies among caregivers of mentally-ill patients. Nurs Midwifery Res J.

[33] Ugargol AP, Bailey A (2018). Family caregiving for older adults: gendered roles and caregiver burden in emigrant households of Kerala. India Asian Popul Stud.

[34] Kadoya Y, Khan MS (2019). Gender differences in the long-term care of older parents: evidence from India. J Fam Stud.

[35] Ugargol AP, Hutter I, James KS, Bailey A (2016). Care needs and caregivers: associations and effects of living arrangements on caregiving to older adults in India. Ageing Int.

[36] International Institute for Population Sciences, MoHFW, HSPH & USC. Longitudinal Ageing Study in India (LASI), Wave 1, 2017–18, India Report. IIPS. 2020; https://www.iipsindia.ac.in/sites/default/files/LASI_India_Report_2020_compressed.pdf

[37] Radloff LS (1977). The CES-D scale: a self-report depression scale for research in the general population. Appl Psychol Meas.

[38] Cohen J. Statistical power analysis for the behavioral sciences. Routledge; 2013.

[39] Vogt WP, Johnson B. Dictionary of statistics & methodology: A nontechnical guide for the social sciences. Sage; 2011.

[40] Aarti, Ruchika, Kumar R, Varghese A. Depression and Quality of Life in Family Caregivers of Individuals with Psychiatric Illness. Int J Community Med Public Health. 2019 Feb; 6(2):715. http://dx.doi.org/10.18203/2394-6040.ijcmph20190196

[41] Ayalon L (2019). Subjective social status as a predictor of loneliness: the moderating effect of the type of long-term care setting. Res Aging.

[42] Dunkle RE, Feld S, Lehning AJ, Kim H, Shen HW, Kim MH (2014). Does becoming an ADL spousal caregiver increase the caregiver’s depressive symptoms?. Res Aging.

[43] Kang SY (2021). Financial strain among unpaid family caregivers of frail elders in community. J Hum Behav Soc Environ.

[44] Lai DW (2012). Effect of financial costs on caregiving burden of family caregivers of older adults. SAGE Open.

[45] Gove WR (1972). The relationship between sex roles, marital status, and mental illness. Soc Forces.

[46] Nieboer AP, Schulz R, Matthews KA, Scheier MF, Ormel J, Lindenberg SM (1998). Spousal caregivers' activity restriction and depression: a model for changes over time. Soc Sci Med.

[47] Glauber R (2017). Gender differences in spousal care across the later life course. Res Aging.

[48] Han SH, Kim K, Burr JA (2021). Activity limitations and depressive symptoms among older couples: the moderating role of spousal care. J Gerontol: Series B.

[49] Berkman LF, Sekher TV, Capistrant B, Zheng Y. Social networks, family, and care giving among older adults in India. In: Smith JP, Majmundar M, editor. Aging in Asia: Findings from new and emerging data initiatives 2012. https://www.ncbi.nlm.nih.gov/sites/books/NBK109207/23077756

[50] Dommaraju P, Visaria A. Gender and ageing in India: Family, intergenerational relationships and the State. In 2017 IPC, IUSSP 2017; https://iussp.confex.com/iussp/ipc2017/meetingapp.cgi/Paper/3835

[51] Funk LM, Chappell NL, Liu G (2013). Associations between filial responsibility and caregiver well-being: are there differences by cultural group?. Res Aging.

[52] Lu N, Lou VW, Zuo D, Chi I (2017). Intergenerational relationships and self-rated health trajectories among older adults in rural China: does gender matter?. Res Aging.

[53] Lindt N, van Berkel J, Mulder BC (2020). Determinants of overburdening among informal carers: a systematic review. BMC Geriatr.

[54] Feld S, Dunkle RE, Schroepfer T, Shen HW (2010). Does gender moderate factors associated with whether spouses are the sole providers of IADL care to their partners?. Res Aging.

[55] Feld S, Dunkle RE, Schroepfer T (2005). When do couples expand their ADL caregiver network beyond the marital dyad?. Marriage Fam Rev.

[56] Weng Y, Li D (2020). Are there benefits to having more children for the oldest-old elderly? a longitudinal analysis on successful aging in China. Asian Popul Stud.

