# Supplementary material for: Caregiving: a risk factor of poor health and depression among informal caregivers in India- A comparative analysis
Source: BMC Public Health. 2023 Jan 6;23:42. doi: 10.1186/s12889-022-14880-5 (PMC9817300; doi:10.1186/s12889-022-14880-5)
Supplement: Supplementary file 1 — Additional file 1: [file 12889_2022_14880_MOESM1_ESM.docx]

| Table 1. Regression Analysis with Associative Factors of Depression and Poor Health Outcome | | | | | | | | |
| --- | --- | --- | --- | --- | --- | --- | --- | --- |
| Background Characteristics | Depressive Symptoms | | | | Poor Self-rated Health | | | |
|  | AOR | p value | Confidence Interval | | AOR | p value | Confidence Interval | |
|  |  |  | Lower | Upper |  |  | Lower | Upper |
|  | | | | | | | | |
| Caregiver |  |  |  |  |  |  |  |  |
| No® |  |  |  |  |  |  |  |  |
| Yes | 1.28 | 0.000 | 1.172 | 1.405 | 1.11 | 0.009 | 1.012 | 1.234 |
|  |  |  |  |  |  |  |  |  |
| Age Group |  |  |  |  |  |  |  |  |
| <45 years ® |  |  |  |  |  |  |  |  |
| 45-59 years | 1.07 | 0.082 | 0.992 | 1.144 | 1.31 | 0.000 | 1.142 | 1.497 |
| 60-69 years | 1.04 | 0.278 | 0.966 | 1.130 | 1.73 | 0.000 | 1.499 | 1.987 |
| >70 years | 1.09 | 0.052 | 0.999 | 1.190 | 2.45 | 0.000 | 2.116 | 2.846 |
|  |  |  |  |  |  |  |  |  |
| Sex |  |  |  |  |  |  |  |  |
| Male ® |  |  |  |  |  |  |  |  |
| Female | 0.94 | 0.012 | 0.903 | 0.988 | 0.86 | 0.000 | 0.808 | 0.924 |
|  |  |  |  |  |  |  |  |  |
| Residence |  |  |  |  |  |  |  |  |
| Rural® | 0.91 | 0.000 | 0.869 | 0.944 | 0.67 | 0.000 | 0.625 | 0.709 |
| Urban |  |  |  |  |  |  |  |  |
|  |  |  |  |  |  |  |  |  |
| Education |  |  |  |  |  |  |  |  |
| No Education ® |  |  |  |  |  |  |  |  |
| Less than 5 years | 1.17 | 0.000 | 1.099 | 1.240 | 0.88 | 0.005 | 0.811 | 0.963 |
| 5-9 years | 0.98 | 0.653 | 0.922 | 1.052 | 0.86 | 0.001 | 0.779 | 0.942 |
| More than 10 years | 0.85 | 0.000 | 0.793 | 0.918 | 0.64 | 0.000 | 0.571 | 0.711 |
|  |  |  |  |  |  |  |  |  |
| Marital Status |  |  |  |  |  |  |  |  |
| Currently Married ® |  |  |  |  |  |  |  |  |
| Widowed | 1.34 | 0.001 | 1.132 | 1.597 | 1.00 | 0.986 | 0.767 | 1.298 |
| Others | 1.16 | 0.121 | 0.960 | 1.413 | 0.89 | 0.435 | 0.654 | 1.200 |
|  |  |  |  |  |  |  |  |  |
| MPCE Quintile |  |  |  |  |  |  |  |  |
| Poorest ® |  |  |  |  |  |  |  |  |
| Poorer | 0.97 | 0.349 | 0.921 | 1.029 | 0.99 | 0.837 | 0.908 | 1.081 |
| Middle | 0.94 | 0.023 | 0.885 | 0.991 | 1.01 | 0.748 | 0.930 | 1.107 |
| Richer | 0.91 | 0.001 | 0.857 | 0.961 | 1.04 | 0.407 | 0.951 | 1.133 |
| Richest | 0.91 | 0.004 | 0.861 | 0.971 | 1.13 | 0.007 | 1.034 | 1.238 |
|  |  |  |  |  |  |  |  |  |
| Caste |  |  |  |  |  |  |  |  |
| SC® |  |  |  |  |  |  |  |  |
| ST | 0.81 | 0.000 | 0.756 | 0.860 | 0.71 | 0.000 | 0.643 | 0.789 |
| OBC | 0.96 | 0.103 | 0.911 | 1.009 | 0.84 | 0.000 | 0.780 | 0.909 |
| None | 0.91 | 0.002 | 0.863 | 0.969 | 0.92 | 0.051 | 0.845 | 1.000 |
|  |  |  |  |  |  |  |  |  |
| Religion |  |  |  |  |  |  |  |  |
| Hindu® |  |  |  |  |  |  |  |  |
| Muslim | 0.99 | 0.672 | 0.931 | 1.047 | 1.19 | 0.000 | 1.093 | 1.291 |
| Others | 0.73 | 0.000 | 0.686 | 0.774 | 0.78 | 0.000 | 0.714 | 0.859 |
|  |  |  |  |  |  |  |  |  |
| Multimorbidity |  |  |  |  |  |  |  |  |
| No Chronic Disease ® |  |  |  |  |  |  |  |  |
| Single Chronic Disease | 1.20 | 0.000 | 1.146 | 1.248 | …. | … | … | … |
| Multimorbidity | 1.47 | 0.000 | 1.397 | 1.543 | … | … | … | … |
|  |  |  |  |  |  |  |  |  |
| Living Arrangement |  |  |  |  |  |  |  |  |
| With Spouse® |  |  |  |  |  |  |  |  |
| With spouse & Children | 0.90 | 0.000 | 0.852 | 0.949 | 1.03 | 0.459 | 0.951 | 1.117 |
| With Children | 0.85 | 0.075 | 0.715 | 1.016 | 1.10 | 0.475 | 0.843 | 1.443 |
| Alone/others | 1.09 | 0.360 | 0.910 | 1.298 | 1.11 | 0.441 | 0.847 | 1.463 |
|  |  |  |  |  |  |  |  |  |
| Social Isolation |  |  |  |  |  |  |  |  |
| Not Isolated® |  |  |  |  |  |  |  |  |
| Isolated | 1.11 | 0.000 | 1.070 | 1.160 | 1.13 | 0.000 | 1.228 | 1.394 |
|  |  |  |  |  |  |  |  |  |
| Satisfaction in Living Arrangement |  |  |  |  |  |  |  |  |
| Satisfied® |  |  |  |  |  |  |  |  |
| Neutral | 2.00 | 0.000 | 1.916 | 2.098 | 1.46 | 0.000 | 1.358 | 1.560 |
| Not Satisfied | 3.68 | 0.000 | 3.402 | 3.978 | 4.13 | 0.000 | 3.750 | 4.538 |
|  |  |  |  |  |  |  |  |  |
| Economic Dependency |  |  |  |  |  |  |  |  |
| Not Dependent® |  |  |  |  |  |  |  |  |
| Dependent | 1.23 | 0.000 | 1.177 | 1.279 | 1.78 | 0.000 | 1.668 | 1.902 |
|  |  |  |  |  |  |  |  |  |
| Consume Tobacco |  |  |  |  |  |  |  |  |
| No® |  |  |  |  |  |  |  |  |
| Yes | 1.01 | 0.746 | 0.964 | 1.052 | 1.25 | 0.000 | 1.168 | 1.328 |
|  |  |  |  |  |  |  |  |  |
| Consume Alcohol |  |  |  |  |  |  |  |  |
| No® |  |  |  |  |  |  |  |  |
| Yes | 0.957 | 0.184 | 0.897 | 1.021 | 0.90 | 0.030 | 0.807 | 0.994 |
|  |  |  |  |  |  |  |  |  |
| Constant | 0.28 | 0.000 | 0.249 | 0.321 | 0.06 | 0.000 | 0.049 | 0.073 |
| Note: SC: Scheduled Caste, ST: Scheduled Tribe, OBC: Other Backward Caste | | | | | | | | |

| Table 2. Prevalence of Depression and Poor Self-rated Health According to Type of Care and Relationship with care Receiver | | | | | | | |
| --- | --- | --- | --- | --- | --- | --- | --- |
| Type of Care | Depressive Symptoms | | | Poor Self-rated Health | | | Total |
|  | Mean | Lower | Upper | Mean | Lower | Upper |  |
| ADL | 0.34 | 0.30 | 0.38 | 0.12 | 0.09 | 0.15 | 1545 |
| IADL | 0.29 | 0.21 | 0.37 | 0.11 | 0.07 | 0.15 | 904 |
| Medical Care | 0.30 | 0.23 | 0.36 | 0.10 | 0.07 | 0.13 | 1124 |
| Social Care | 0.34 | 0.29 | 0.39 | 0.15 | 0.10 | 0.19 | 1080 |
| Financial Care | 0.37 | 0.31 | 0.43 | 0.11 | 0.08 | 0.14 | 658 |
|  |  |  |  |  |  |  |  |
| Relationship with Receiver | | | | | | | |
| Spouse/Partner | 0.30 | 0.19 | 0.42 | 0.12 | 0.07 | 0.18 | 588 |
| Parents | 0.32 | 0.25 | 0.38 | 0.10 | 0.04 | 0.16 | 503 |
| Parents-in-law | 0.22 | 0.15 | 0.28 | 0.08 | 0.03 | 0.13 | 369 |
| Brother/Sister | 0.44 | 0.22 | 0.65 | 0.25 | 0.06 | 0.45 | 62 |
| Children | 0.38 | 0.28 | 0.48 | 0.15 | 0.09 | 0.21 | 314 |
| Other relatives | 0.31 | 0.23 | 0.39 | 0.11 | 0.05 | 0.17 | 245 |
| Not related | 0.39 | 0.24 | 0.55 | 0.17 | 0.00 | 0.34 | 143 |
| Note: ADL: Activities in daily living, IADL: Instrumental Activities in daily living | | | | | | | |
